# Comparison of bone regenerative capacity of donor-matched human adipose–derived and bone marrow mesenchymal stem cells

**DOI:** 10.1007/s00441-020-03315-5

**Published:** 2020-11-26

**Authors:** Samih Mohamed-Ahmed, Mohammed A. Yassin, Ahmad Rashad, Heidi Espedal, Shaza B. Idris, Anna Finne-Wistrand, Kamal Mustafa, Hallvard Vindenes, Inge Fristad

**Affiliations:** 1 Department of Clinical Dentistry, Faculty of Medicine, University of Bergen, Bergen, Norway; 2 Department of Biomedicine, University of Bergen, Bergen, Norway; 3 Department of Fibre and Polymer Technology, KTH Royal Institute of Technology, Stockholm, Sweden; 4 Department for Plastic, Hand and Reconstructive Surgery, National Fire Damage Center, Bergen, Norway

**Keywords:** Adipose-derived stem cell, Bone marrow mesenchymal stem cell, Osteogenic differentiation, Calvarial defect, Bone regeneration

## Abstract

Adipose-derived stem cells (ASC) have been used as an alternative to bone marrow mesenchymal stem cells (BMSC) for bone tissue engineering. However, the efficacy of ASC in bone regeneration in comparison with BMSC remains debatable, since inconsistent results have been reported. Comparing ASC with BMSC obtained from different individuals might contribute to this inconsistency in results. Therefore, this study aimed to compare the bone regenerative capacity of donor-matched human ASC and BMSC seeded onto poly(l-lactide-co-ε-caprolactone) scaffolds using calvarial bone defects in nude rats. First, donor-matched ASC and BMSC were seeded onto the co-polymer scaffolds to evaluate their in vitro osteogenic differentiation. Seeded scaffolds and scaffolds without cells (control) were then implanted in calvarial defects in nude rats. The expression of osteogenesis-related genes was examined after 4 weeks. Cellular activity was investigated after 4 and 12 weeks. Bone formation was evaluated radiographically and histologically after 4, 12, and 24 weeks. In vitro, ASC and BMSC demonstrated mineralization. However, BMSC showed higher alkaline phosphatase activity than ASC. In vivo, human osteogenesis–related genes Runx2 and collagen type I were expressed in defects with scaffold/cells. Defects with scaffold/BMSC had higher cellular activity than defects with scaffold/ASC. Moreover, bone formation in defects with scaffold/BMSC was greater than in defects with scaffold/ASC, especially at the early time-point. These results suggest that although ASC have the potential to regenerate bone, the rate of bone regeneration with ASC may be slower than with BMSC. Accordingly, BMSC are more suitable for bone regenerative applications.

## Introduction

Repair of skeletal defects resulting from trauma, degenerative diseases, and tumor resection remains a medical challenge. Cell-based tissue engineering using mesenchymal stem cells (MSC) has emerged as a new approach for regeneration of damaged skeletal tissues. MSC are undifferentiated multipotent cells of mesenchymal origin with self-renewal capacity and potential to differentiate into cells of mesenchymal origin when exposed to specific growth signals (Shanti et al. 2007). MSC play a central role in maintenance and regeneration of body tissues, and these cells can be isolated from different body organs and tissues (Beane and Darling 2012). Bone marrow MSC (BMSC) have been widely used in tissue engineering (Marolt et al. 2010). However, limited amounts of MSC exist in bone marrow, as they represent only 0.001–0.01% of the nucleated cells (Pittenger et al. 1999). This has led to an increased interest in MSC from other sources, especially adipose tissue. Adipose-derived stem cells (ASC) exist in large amounts and can be isolated from adipose tissue from multiple sites with minimum discomfort for the patients (Fraser et al. 2006; Raposio et al. 2016). ASC share morphology and immunophenotype characteristics with BMSC, and like BMSC, ASC have multilineage differentiation capacity and a great potential for regenerative applications (Mizuno et al. 2012).

For bone tissue engineering (BTE) applications, the efficacy of BMSC in regenerating bone has been investigated in many in vivo studies (Dang et al. 2017; Kanczler et al. 2008; Koob et al. 2011; Zong et al. 2010). Similarly, the in vivo bone regenerative capacity of ASC has been studied (Levi et al. 2010; Peña González et al. 2016; Yoon et al. 2007). Previous studies have also compared the in vivo capacity of ASC and BMSC in bone regeneration to evaluate the effect of these MSC for BTE (Bothe et al. 2018; Degano et al. 2008; Freitas et al. 2019; Jo et al. 2013; Kang et al. 2012; Kargozar et al. 2018; Kim et al. 2014; Lin et al. 2009; Niemeyer et al. 2010; Stockmann et al. 2012; Walmsley et al. 2016; Wen et al. 2013; Xu et al. 2017). Many of these studies reported similar bone regenerative ability of ASC and BMSC (Degano et al. 2008; Freitas et al. 2019; Jo et al. 2013; Kang et al. 2012; Kim et al. 2014; Lin et al. 2009; Stockmann et al. 2012; Walmsley et al. 2016; Wen et al. 2013), whereas a greater in vivo capacity of BMSC in bone regeneration was reported in other studies (Bothe et al. 2018; Kargozar et al. 2018; Lin et al. 2009; Niemeyer et al. 2010; Xu et al. 2017). However, only a limited number of these in vivo studies compared human ASC and BMSC (Bothe et al. 2018; Degano et al. 2008; Jo et al. 2013; Kargozar et al. 2018; Kim et al. 2014; Wen et al. 2013; Xu et al. 2017), and most of these studies compared human ASC and BMSC from different individuals, which might influence the results (Mohamed-Ahmed et al. 2018). Therefore, comparing the in vivo bone regenerative capacity of donor-matched human BMSC and ASC should provide more reliable results.

Scaffold, which act as a carrier and provide structural support for cells, must be osteoconductive to be used in BTE. This means that bone cells can adhere to the scaffold, produce extracellular matrix (ECM), and eventually form bone on the surface and inside the pores of the scaffolds (Albrektsson and Johansson 2001). Synthetic polymers have been used for scaffold production (Hutmacher 2000). Among different polymers, poly(l-lactide-co-ε-caprolactone) (poly(LLA-co-CL)) scaffolds have been extensively investigated by our group, both in vitro and in vivo, and their suitability for BTE applications has been demonstrated (Dånmark et al. 2010; Idris et al. 2010a, 2010b; Xing et al. 2013; Yassin et al. 2017). In a previous study, we compared donor-matched ASC and BMSC in terms of proliferation and differentiation under two-dimensional (2D) conditions (Mohamed-Ahmed et al. 2018). However, 2D conditions do not represent the in vivo three-dimensional (3D) environment (Fitzgerald et al. 2015). Therefore, the aim of this study was to compare the bone regenerative capacity of donor-matched human ASC and BMSC seeded onto poly(LLA-co-CL) scaffolds using critical-size calvarial bone defects in nude rats.

## Material and methods

### Isolation and expansion of human ASC and BMSC

Subcutaneous adipose tissues and bone marrow aspirates were obtained from three young donors (female 8 years; males 9 and 12 years) at the Department for Plastic, Hand, and Reconstructive Surgery, National Fire Damage Center, Bergen, Norway, with informed parental consent. ASC were isolated as previously described (Mohamed-Ahmed et al. 2018). In brief, after washing with phosphate-buffered saline (PBS) (Invitrogen, Carlsbad, CA, USA) with 5% antibiotics (penicillin/streptomycin; GE Healthcare Life Sciences, South Logan, UT, USA), adipose tissue was digested with 0.1% collagenase type I (Worthington Biochemical Corporation Lakewood, NJ, USA) in PBS for 60 min. An equal amount of culture medium (Dulbecco’s modified Eagle’s medium (DMEM) (Invitrogen) with 10% fetal bovine serum (FBS) (Hyclone, GE Healthcare Life Sciences) and 1% antibiotics) was added to neutralize the collagenase before centrifugation at 2000 rpm for 5 min. Supernatant was removed and the pellet was suspended in culture medium and cultured in a 75-cm^2^ culture flask (NUNC™, Thermo Fisher Scientific, Waltham, MA, USA). BMSC were isolated as previously described (Mohamed-Ahmed et al. 2018). In brief, after filtering the aspirate with a 70-μm cell strainer (Fisher Scientific, Hampton, NH, USA), the aspirate was diluted with an equal amount of culture medium and then centrifuged at 1800 rpm for 10 min. The supernatant was removed and the cell pellet was suspended in culture medium and cultured in a 75-cm^2^ culture flask. ASC and BMSC were incubated under humidified conditions at 37 °C with 5% CO_2_. Cells were then washed with PBS after 24 h, before culture medium again was added, and then changed twice a week. BMSC and ASC were subcultured at a density of 5 × 10^3^ cells/cm^2^, and cells at passage 4 were used in this study.

### Characterization of ASC and BMSC

ASC and BMSC were characterized based on expression of the surface markers, CD34, CD45, CD73, CD90, CD105, HLA-DR (BD Biosciences, San Jose, CA, USA), and Stro-1 (Santa Cruz Biotechnology, Dallas, TX, USA) according to manufacturer’s recommendations. Samples without monoclonal antibodies were used as control. Flow cytometry was performed in a BD LSRFortessa Cell Analyzer (BD Biosciences). Flow cytometry data were analyzed using analysis software (FlowJo V10, Flowjo, LLC, Ashland, OR, USA).

### Scaffold fabrication and preparation

Poly(LLA-co-CL) scaffolds were fabricated using a solvent casting-particulate leaching method as previously described (Danmark et al. 2011). Briefly, required amounts of monomer, initiator, and catalysts were bulk polymerized for 72 h at 110 °C in an inert atmosphere. The formed copolymer was precipitated in cold hexane and methanol three times. The copolymer was dissolved in chloroform and poured into a glass mold containing sodium chloride. After evaporation of the solvent, salt particles were leached by soaking in deionized water and the salt-free scaffolds were vacuum dried. The scaffolds were porous with 90–500 μm pore size and high interconnectivity. For sterilization, scaffolds were exposed to a dose of 2.5 Mrad electron beam radiation from a pulsed electron accelerator (Mikrotron, Acceleratorteknik, Stockholm, Sweden) at 6.5 meV, in an inert atmosphere (Danmark et al. 2011). Porous scaffolds of 5 mm diameter and 1.2 mm thickness were placed in a 96-well plate (NUNC™, Thermo Fisher Scientific, Waltham, MA, USA) and pre-wetted overnight with culture medium under humidified conditions at 37 °C with 5% CO_2_ before seeding of cells.

### Cell attachment and in vitro proliferation

ASC and BMSC were seeded onto scaffolds at a seeding density of 1 × 10^5^ cells/scaffold for the in vitro experiments. Preparations of scaffold/ASC and scaffold/BMSC were incubated under humidified conditions at 37 °C with 5% CO_2_. To investigate attachment of cells after 1 day, scaffold/ASC and scaffold/BMSC were fixed in 3% glutaraldehyde (Merck, Readington, NJ, USA), dehydrated in graded ethanol solutions, vacuum dried, and sputter-coated with platinum. Scaffolds were then imaged using a scanning electron microscope (SEM) (Jeol, Tokyo, Japan) at 5 kV. After 1 day of seeding, seeded scaffolds were cultured in osteogenic medium (culture medium supplemented with 0.05 mM l-ascorbic acid 2-phosphate, 10 nM dexamethasone, and 10 mM β glycerophosphate (Sigma-Aldrich)). In vitro cell proliferation was evaluated at days 1, 7, 10, and 14 using Quant-iT™ PicoGreen™ dsDNA Assay Kit (Invitrogen). Cells were lysed with 200 µl of 0.1% Triton-X100 buffer (Sigma-Aldrich, St. Louis, MO, USA), followed by two freezing-thawing cycles at − 80 °C. Equal amounts of lysate solution and PicoGreen dye were added into a 96-well plate, and fluorescence intensity was measured using a microplate reader (FLUOstar OPTIMA, BMG Labtech, Offenburg, Germany) at 485 nm excitation and 525 nm emission. A DNA standard curve was made using solutions with known DNA concentration.

### Evaluation of in vitro osteogenic differentiation

To evaluate the in vitro osteogenic capacity of ASC and BMSC, culture medium was replaced with osteogenic medium 1 day after seeding. As control, seeded scaffolds were cultured in normal culture medium. Alkaline phosphatase (ALP) was measured using *p*-Nitrophenyl Phosphate Liquid Substrate System (Sigma-Aldrich) at days 3, 7, and 14. Samples were lysed in 0.1% Triton-X100 buffer, followed by two freezing-thawing cycles at − 80 °C. Equal amounts of lysate solution and *p*-Nitrophenyl Phosphate Liquid Substrate were added into a 96-well plate, incubated for 30 min at 37 °C, and absorbance was measured using the microplate reader at 405 nm. To assess calcium deposition after 21 days, scaffold/cells were fixed with 4% paraformaldehyde (Merck), stained with 2% Alizarin red S (Sigma-Aldrich) for 30 min, washed, and left to dry overnight. The stain was dissolved in cetylpyridinium chloride (Sigma-Aldrich) for quantification using a microplate reader at 540 nm absorbance.

### In vivo experiment design

ASC and BMSC were cultured in osteogenic medium for 4 days before seeding onto scaffolds at a density of 1 × 10^6^ cells/scaffold. The scaffolds were then shaken on an orbital shaker (Eppendorf, Hamburg, Germany) for 30 s. Scaffold/ASC and scaffold/BMSC were incubated overnight under humidified conditions at 37 °C with 5% CO_2_ before implantation. Twenty-six 10-week-old female nude rats, weighing 250–300 g, were anesthetized with sevoflurane (SevoFlo®, Abbott Laboratories Ltd, Berkshire, UK) and O_2_ gas mixture using a custom-made mask. The surgical site was shaved and scrubbed with chlorhexidine (HiBiSCRUB®, Regent Medical Ltd, Lancashire, UK) before making a 2-cm sagittal incision in the midline using a sterile scalpel (B. Braun, Melsungen, Germany). Calvaria were exposed after dissection and periosteal elevation. Two defects, 5 mm diameter (one defect in each parietal bone), were carefully created in each rat using a saline-cooled trephine drill (Hager & Meisinger GmbH, Neuss, Germany), leaving the dura mater undamaged. Scaffold/ASC, scaffold/BMSC, and pre-wetted scaffolds without cells (control) were randomly implanted in the 52 defects. The periosteum and skin were sutured with interrupted stitches (VICRYL®, Ethicon, Somerville, NJ, USA). The rats were injected subcutaneously with Buprenorphine (Temgesic 0.3 mg/kg, Indivior UK LTD, Berkshire, UK) as postoperative analgesia. After recovery from anesthesia, the health of the rats was regularly monitored. The rats were euthanized by an overdose of CO_2_ at weeks 4 and 24, and the calvaria were harvested and kept in RNAlater (Invitrogen) at − 80 °C for further investigations.

### Real-time quantitative polymerase chain reaction (qPCR)

RNA was extracted from the week 4 samples using a Maxwell® 16 LEV simplyRNA kit (Promega, Madison, WI, USA). RNA amount and purity were measured using Nanodrop ND-1000 Spectrophotometer (Nanodrop Technologies, Wilmington, DE, USA). cDNA was synthesized using a High-Capacity cDNA Reverse Transcription Kit (Applied Biosystems, Foster City, CA, USA). Real-time qPCR, using TaqMan Fast Universal PCR Master Mix (Applied Biosystems), was completed using a Stepone™ Real-Time PCR System (Applied Biosystems). The expression of the osteogenesis-related human genes runt-related transcription factor 2 (Runx2) (Hs00298328_s1) and collagen type I (Hs00164099_m1) and rat genes Runx2 (Rn01512298_m1) and collagen type I (Rn01463848_m1) was detected. Human (Hs02758991_g1) and rat (Rn01749022_g1) glyceraldehyde-3-phosphate dehydrogenase (GAPDH) genes were used as an endogenous control. All primers were from Applied Biosystems. The expression of the rat genes was presented relative to control (scaffolds without cells) while the expression of the human genes was presented relative to scaffold/BMSC. Data were analyzed by the 2^−Δ∆CT^ method.

### Immunofluorescence staining of the human nuclei 

Sections from the week 4 samples were processed for immunofluorescence staining of the human nuclei. The sections were washed twice with 0.1% Tween 20 (Sigma-Aldrich) in PBS (TPBS) for 5 min and then blocked for 30 min at room temperature with 10% normal goat serum (Dako, Glostrup, Denmark) in TPBS. After blocking, the sections were incubated overnight at 4 °C with mouse monoclonal anti-nuclei antibody (MAB1281, Millipore, Temecula, CA, USA; dilution 1:20) in TPBS with 1% goat serum. After two washes with TPBS for 5 min, the sections were incubated for 45 min at room temperature with goat anti-mouse IgG secondary antibody in TBST (Alexa Fluor 488, Life Technologies, Carlsbad, CA, USA; dilution 1:250). The sections were then washed twice with PBS and stained at room temperature with 4′,6-diamidino-2-phenylindole (DAPI) (Sigma-Aldrich; dilution 1:2000) for 5 min. After washing with PBS, images were taken using an inverted fluorescent microscope (Nikon Eclipse Ti, Tokyo, Japan).

### Positron emission tomography/computed tomography (PET/CT) imaging

Rats were subjected to 18F-sodium fluoride (^(18F)^NaF) PET/CT in vivo imaging on a nanoScan small animal scanner (Mediso Medical Imaging System Budapest, Hungary) at weeks 4 and 12. During anesthesia, each rat was injected with the radioactive tracer (≈ 13.5 ^(18F)^NaF MBq in saline solution − total volume 1 ml) through the tail vein. After 40 min, a PET emission scan of 20 min was performed to measure the uptake of the tracer. For attenuation correction of the PET images, a CT scan of the same anatomical volume as the PET scan was acquired (voxel size 125 × 125 × 250 μm, energy 50 kV, exposure time 300 ms, projections 480, and binning 1:4). CT scanning was also used for analysis of bone formation in the defects (voxel size 20 × 20 × 20 μm, energy 70 kV, exposure time 300 ms, projections 720, and binning 1:1) by evaluating bone density (BD) and bone volume (BV). PET and CT images were reconstructed on a dedicated Mediso workstation. Data were analyzed using PMOD software (PMOD Technologies LLC, Zurich, Switzerland). Volume-of-interest (VoI) was manually drawn for each bone defect and was used for both the PET and CT quantifications. For the PET, the standardized uptake value (SUV) mean, within the VoI, was quantified. For the bone formation quantification, an isocontour with a threshold of 741 HU within the VoI was applied. The threshold was determined by scanning a dedicated bone mineral density phantom (Bruker microCT, Kontich, Belgium). Bone formation was quantified and presented as BV normalized to BD.

### Micro-CT scanning

Rat calvaria at weeks 4 and 24 were fixed in 4% paraformaldehyde for 24 h. Micro-CT scanning of the calvaria was performed using SkyScan1172 (Bruker microCT) for quantification of new bone formation (Vo et al. 2015). Samples were scanned with an X-ray source of 60 kV/200 µA, a 0.5-mm aluminum filter for a 10-µm resolution, and a 0.4° rotation step. The projection image was reconstructed using NRecon ReconstructionVR CT software (Bruker microCT). The quantitative analysis of the image was performed by CTan software (Bruker microCT). A global threshold of 90–255 was applied to all calvaria after determining the standardized cylindrical VoI, 5 mm in diameter and 1.3 mm in height. Data were reported as the percentage binarized object volume measured within this VoI, defined as bone volume (BV), tissue volume (TV), and BV/TV.

### Histological analysis

After scanning, the rat calvaria harvested at weeks 4 and 24 were maintained in 10% ethylenediaminetetraacetic acid (EDTA) solution (Merck) for 4 weeks for decalcification. Calvaria were then cut into two halves. Each defect was identified and embedded in paraffin. Serial sections of 6 µm were stained with H and E.

### Statistical analysis

For statistical analysis, a two-tail Student’s *t* test was applied to determine the statistical significance of the differences between the groups. The results are presented as mean ± SD. *P* values < 0.05 were considered statistically significant and are indicated in the figures by an asterisk.

## Results

### Morphologic and immunophenotypic characterization of ASC and BMSC

**Fig. 1:**
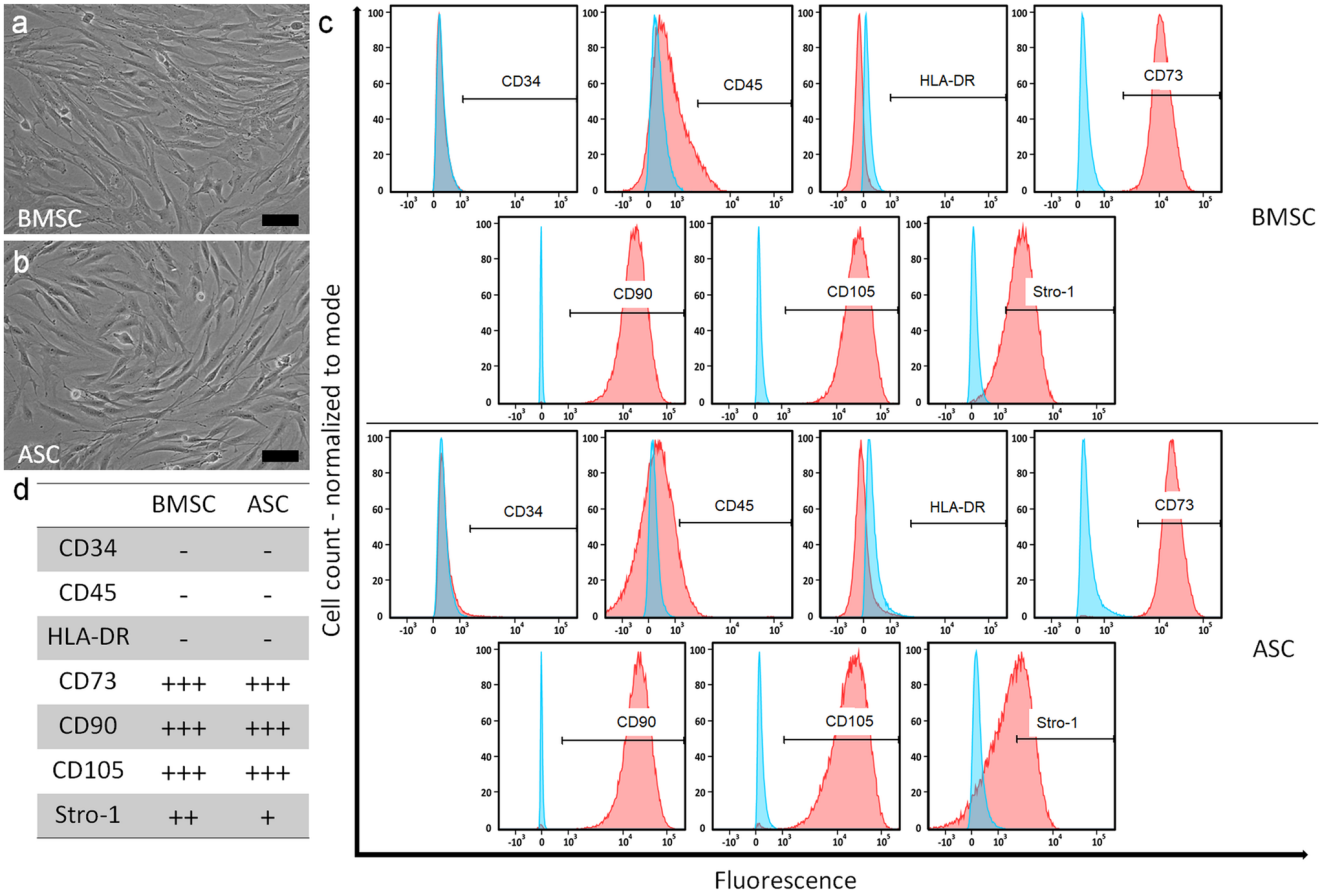
Morphology and immunophenotype characteristics of BMSC and ASC. **a**, **b** Representative microscopic images of BMSC and ASC; scale bar 100 µm. **c** Histograms showing flow cytometry analysis of BMSC and ASC, antibody control (blue) and the stained cells (red). **d** Surface marker expression on BMSC and ASC. Dash, ≤ 10% expression; single plus sign, 11–50% expression; double plus sign, 51–90% expression; and triple plus sign, > 90% expression. BMSC, bone marrow mesenchymal stem cells; ASC, adipose-derived stem cells

ASC and BMSC, adhering to the plastic culture flask, demonstrated a fibroblast-like morphology (Fig. 1 a and b). ASC and BMSC were expanded up to passage 4 without visible morphologic changes. ASC and BMSC demonstrated high expression of the surface markers CD73, CD90, and CD105. The negative expression of the surface markers CD34, CD45, and HLA-DR was seen for both ASC and BMSC. ASC and BMSC demonstrated the expression of Stro-1, but the expression was higher in BMSC (Fig. 1 c and d).

### Cell attachment and proliferation on the poly(LLA-co-CL) scaffolds

**Fig. 2:**
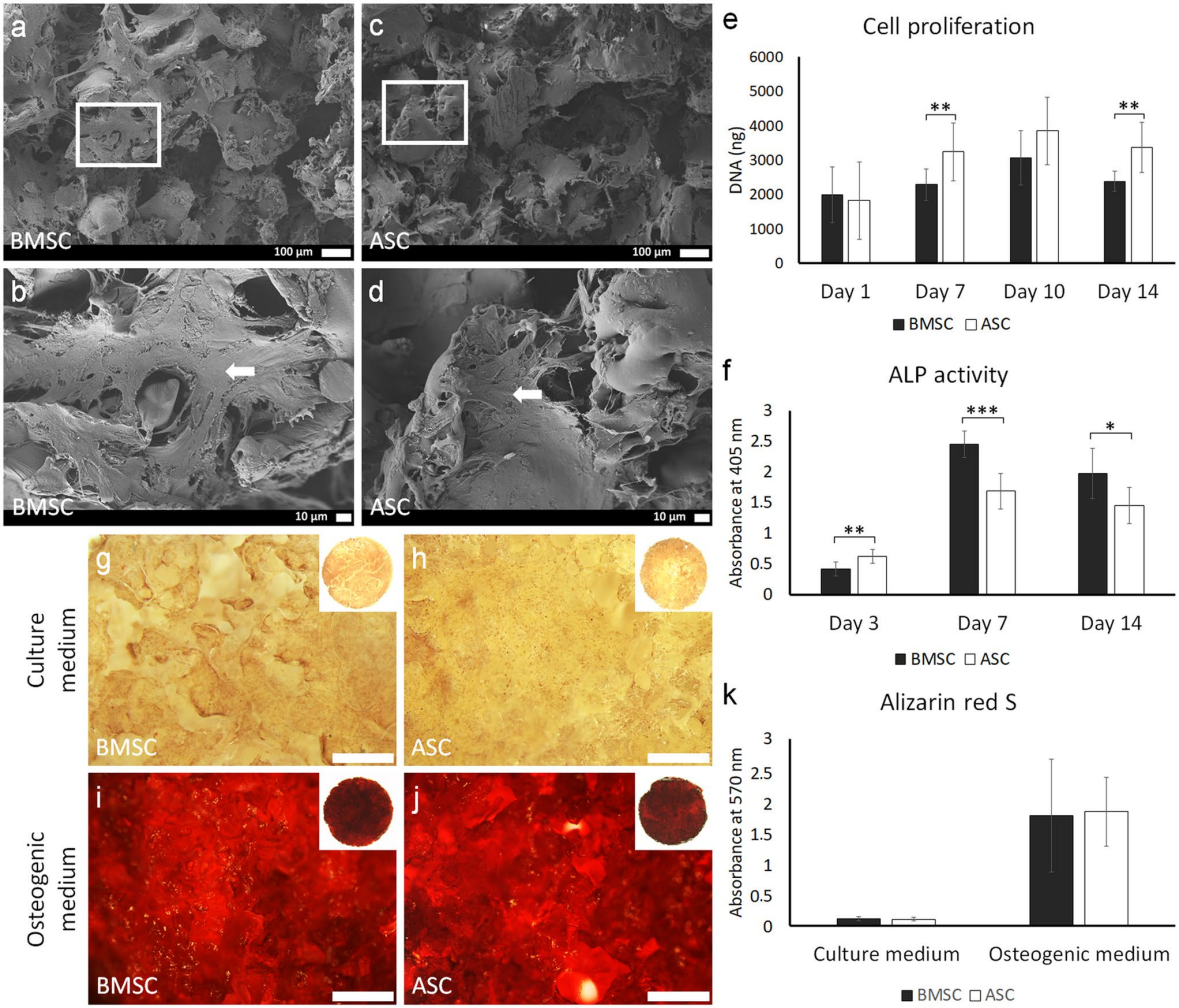
BMSC and ASC attachment, proliferation, and osteogenic differentiation on scaffolds. **a**–**d** SEM images of attached BMSC and ASC on the scaffold at day 1; scale bars 100 µm and 10 µm. White arrows indicate cell sheets. **e** Proliferation of BMSC and ASC on the scaffold at days 1, 7, 10, and 14. **f** ALP activity assay of BMSC and ASC on the scaffold at days 3, 7, and 14. **g**–**j** Representative images of Alizarin red S staining of BMSC and ASC on the scaffold at day 21; scale bar 100 µm. **k** Graph with quantitative data of Alizarin Red S staining. BMSC, bone marrow mesenchymal stem cells; ASC, adipose-derived stem cells; SEM, scanning electron microscope; ALP, alkaline phosphatase. *p < 0.05; **p < 0.01; ***p < 0.001

The attachment of ASC and BMSC on the poly(LLA-co-CL) scaffolds was confirmed 1 day after seeding. ASC and BMSC were attached and spread on the scaffold, as confirmed by scanning electron microscope (SEM) images (Fig. 2a–d). ASC and BMSC proliferated on the scaffolds from day 1 to 10; then, the proliferation decreased at day 14. This decreased proliferation was statistically significant for BMSC (*p* < 0.05), but not ASC. Greater amount of DNA was detected in scaffolds seeded with ASC compared with scaffolds seeded with BMSC, reaching significance at days 7 and 14 (*p* < 0.01) (Fig. 2e).

### In vitro osteogenic capacity

ASC and BMSC seeded on the scaffolds in osteogenic medium showed in vitro osteogenic capacity, confirmed by ALP activity at days 3, 7, and 14, and Alizarin red S staining at day 21. ASC and BMSC showed a similar trend in ALP activity up to day 14. At day 3, the ALP activity in ASC was significantly higher than in BMSC (*p* < 0.01) (Fig. 2f). However, at days 7 and 14, BMSC showed significantly higher ALP activity than ASC (*p* < 0.05). Both BMSC and ASC showed the highest ALP activity at day 7. No differences in the in vitro mineralization were detected between ASC and BMSC (Fig. 2g–k). ASC and BMSC in control culture medium did not show signs of mineralization.

### In vivo gene expression and human nuclei staining

**Fig. 3:**
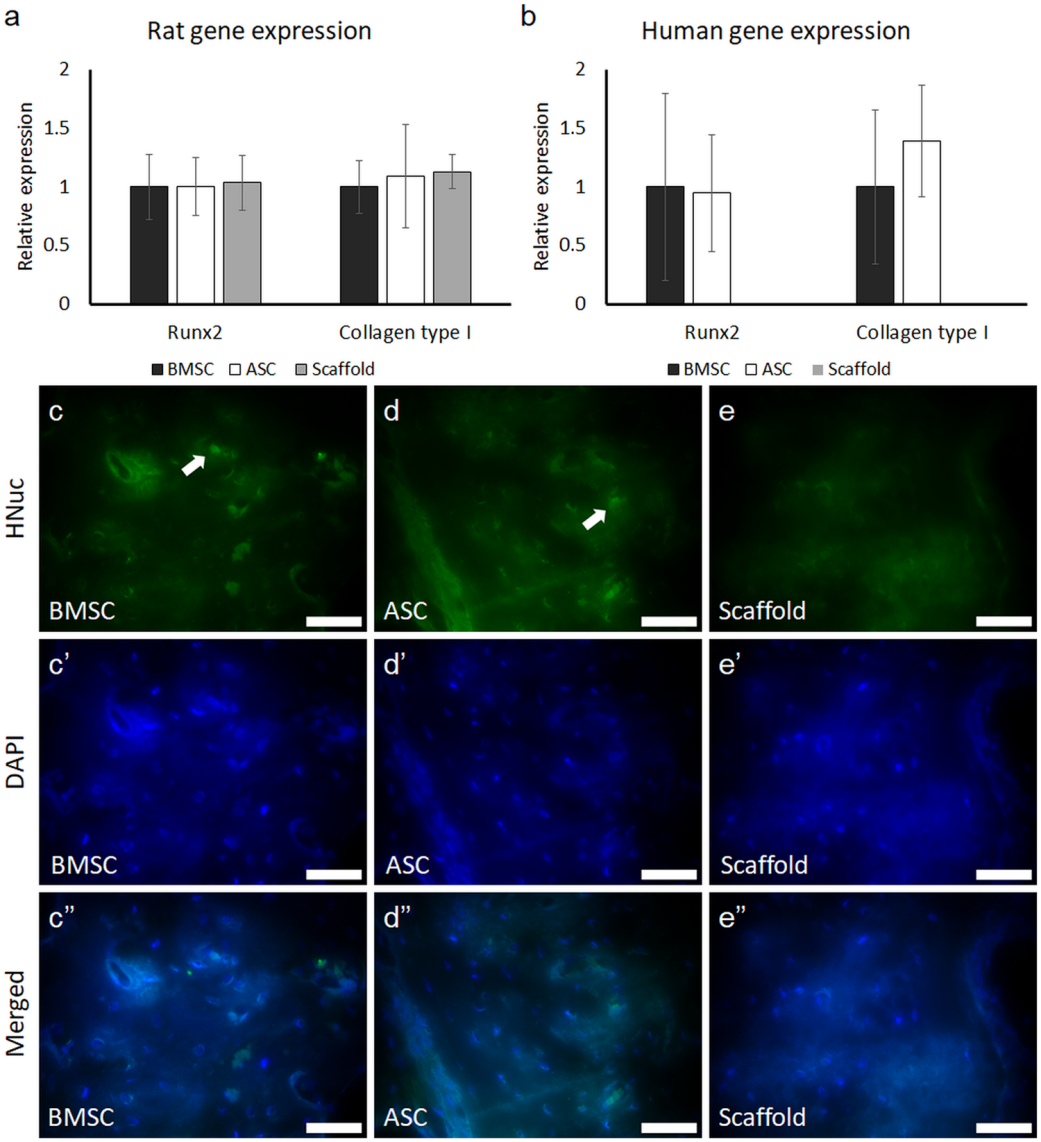
Expression of the osteogenesis-related genes and human nuclei staining in defects with scaffold/BMSC, scaffold/ASC, and scaffold without cells after in vivo implantation for 4 weeks. **a** Rat gene expression. **b** Human gene expression. **c**–**e** Immunofluorescence staining of human nuclei in the newly formed bone; scale bar 50 μm. BMSC, bone marrow mesenchymal stem cells; ASC, adipose-derived stem cells; Runx2, runt-related transcription factor 2; HNuc, human nuclei; DAPI 4′,6-diamidino-2-phenylindole

The expression of the human and rat osteogenesis–related genes Runx2 and collagen type I was detected at week 4 (Fig. 3 a and b). The rat genes Runx2 and collagen type I were expressed in defects treated with scaffold/ASC, scaffold/BMSC, or scaffold without cells. No differences in the expression of the rat genes were detected among the three groups. Defects treated with scaffold/ASC or scaffold/BMSC showed the expression of the human genes Runx2 and collagen type, while, as expected, the control defects showed no expression of these human genes. There were no differences in the expression of the human genes Runx2 and collagen type I between the defects treated with scaffold/ASC or scaffold/BMSC. Immunofluorescence staining showed human nuclei embedded in the newly formed bone in defects treated with scaffold/ASC or scaffold/BMSC after 4 weeks (Fig. 3 c and d). No signal of human nuclei was detected in the control defects (Fig. 3e).

### Cellular activity and bone formation

**Fig. 4:**
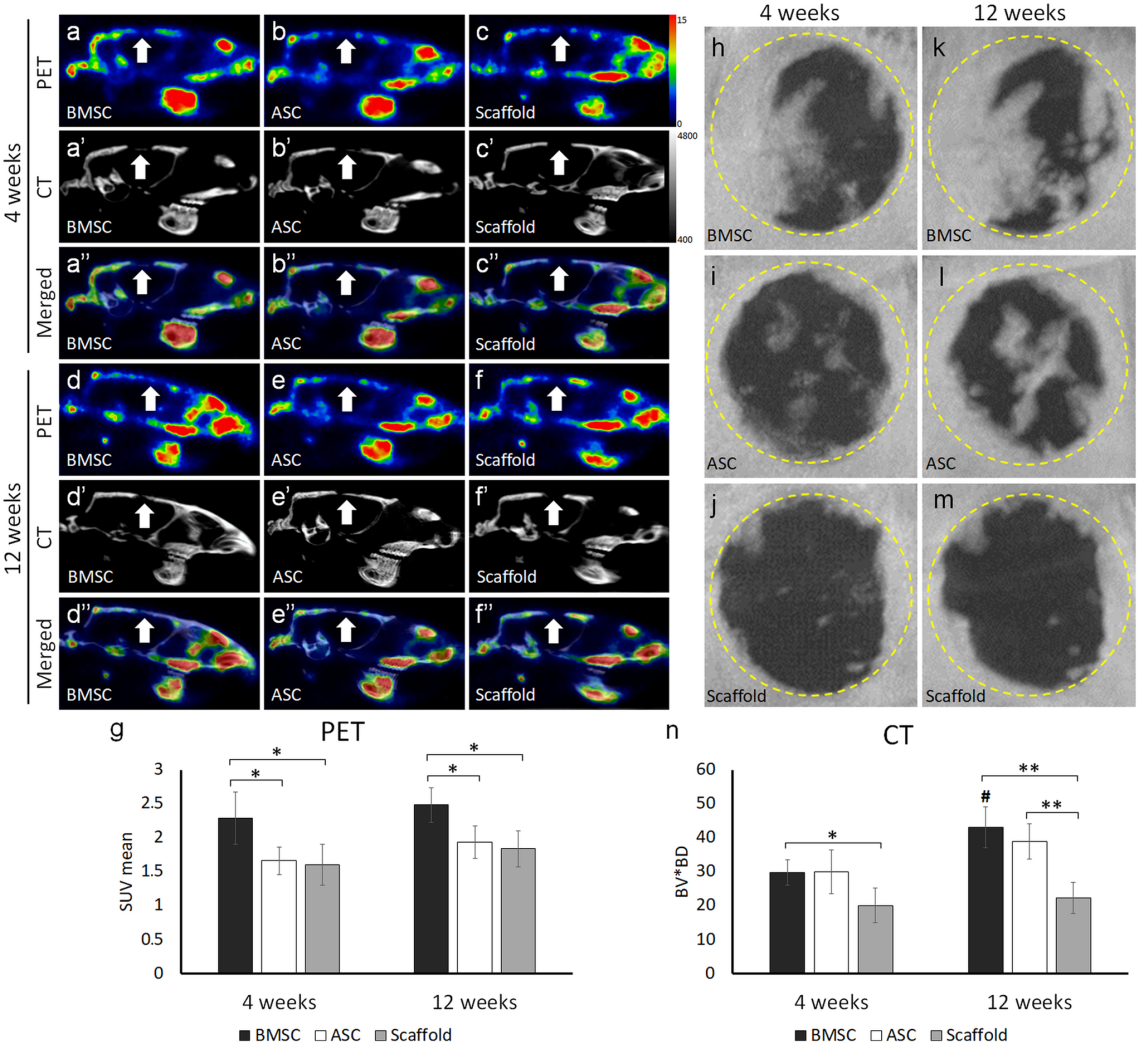
Osteogenic cellular activity and bone formation in defects with scaffold/BMSC, scaffold/ASC, and scaffold without cells after in vivo implantation for 4 and 12 weeks. **a**–**f** Representative images of sagittal sections from the PET/CT scanning. White arrows indicate defect area. **g** Quantitative graph based on the PET data. **h**–**m** Representative CT scanning images showing bone formation in the defects. **n** Quantitative graph based on the CT data. BMSC, bone marrow mesenchymal stem cells; ASC, adipose-derived stem cells; PET/CT, positron emission tomography/computed tomography; SUV, standardized uptake value; BV, bone volume; BD, bone density. Number sign indicates a significant difference (*p* < 0.05) between different time-points of the same group. *p < 0.05; ** *p* < 0.01

Cellular activity and bone formation in the defects were investigated using PET/CT imaging 4 and 12 weeks postoperatively. At week 4, the SUV mean of the tracer uptake in the defects with scaffold/BMSC was higher than in the defects with scaffold/ASC or control (*p* < 0.05) (Fig. 4a–g). At week 12, the uptake tended to increase compared with week 4 in all defects, but as seen at week 4, the defects with scaffold/BMSC showed significantly higher uptake than defects with scaffold/ASC or control (*p* < 0.05). The CT analysis at week 4 showed that new bone started to form in all the defects (Fig. 4h–n). However, greater amount of bone was formed in defects with scaffold/cells compared with control, reaching significance level for scaffold/BMSC (*p* < 0.05). At week 12, the amount of bone was increased in the scaffold/cells groups. This was significant in defects with scaffold/BMSC (*p* < 0.05), but not in defects with scaffold/ASC. The defects with scaffold/BMSC or scaffold/ASC showed significantly greater bone formation than the control defects (*p* < 0.01). The defects treated with scaffold/BMSC had the greatest amount of bone formation.

### Bone formation evaluated by micro-CT

**Fig. 5:**
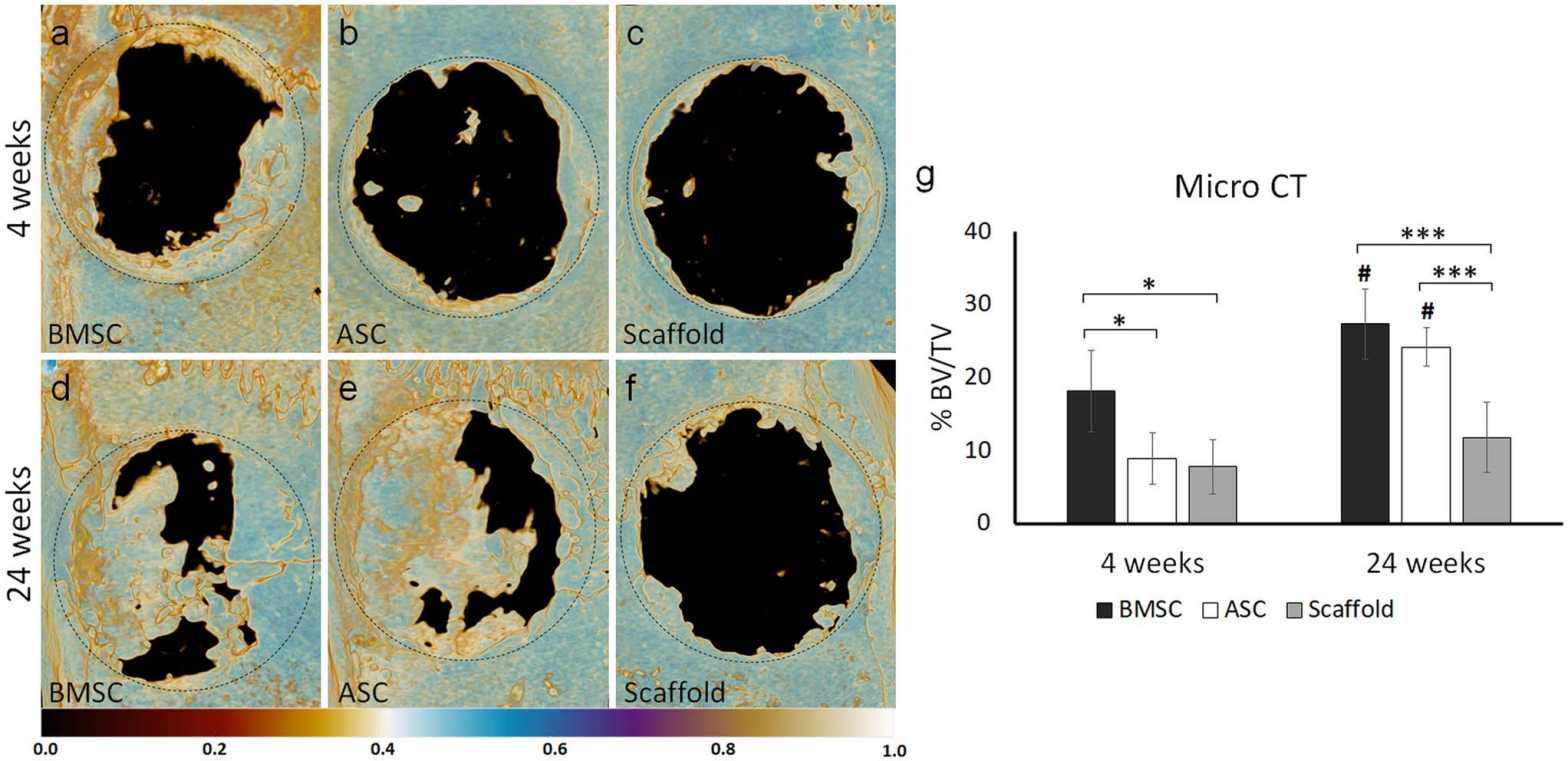
Micro-CT analysis of bone formation in defects with scaffold/BMSC, scaffold/ASC, and scaffold without cells after in vivo implantation for 4 and 24 weeks. **a**–**f** Representative images showing bone formation. **g** Graph showing quantitative data based on the analysis. BMSC, bone marrow mesenchymal stem cells; ASC, adipose-derived stem cells; CT, computed tomography; BV, bone volume; TV, tissue volume. Number sign indicates a significant difference (*p* < 0.05) between different time-points of the same group. *p < 0.05; ***p < 0.001

Bone formation was evaluated using micro-CT 4 and 24 weeks postoperatively (Fig. 5). At week 4, the mean percentage of BV/TV in the defects treated with scaffold/BMSC was 18.1 ± 5.6%, significantly greater than the defects with scaffold/ASC or control with 8.9 ± 3.5% and 7.8 ± 3.7%, respectively (*p* < 0.05). At week 24, the BV/TV was significantly increased to 27.4 ± 4.8% in defects with scaffold/BMSC and 24.2 ± 2.7% in defects treated with scaffold/ASC compared with week 4 (*p* < 0.01). The increase of BV/TV in defects treated with control scaffolds to 11.8 ± 4.8% was not statistically significant. The BV/TV in defects treated with scaffold/BMSC or scaffold/ASC was significantly higher than the control defects (*p* < 0.001). The highest BV/TV was observed in the defects treated with scaffold/BMSC.

### Histological evaluation of bone formation

**Fig. 6:**
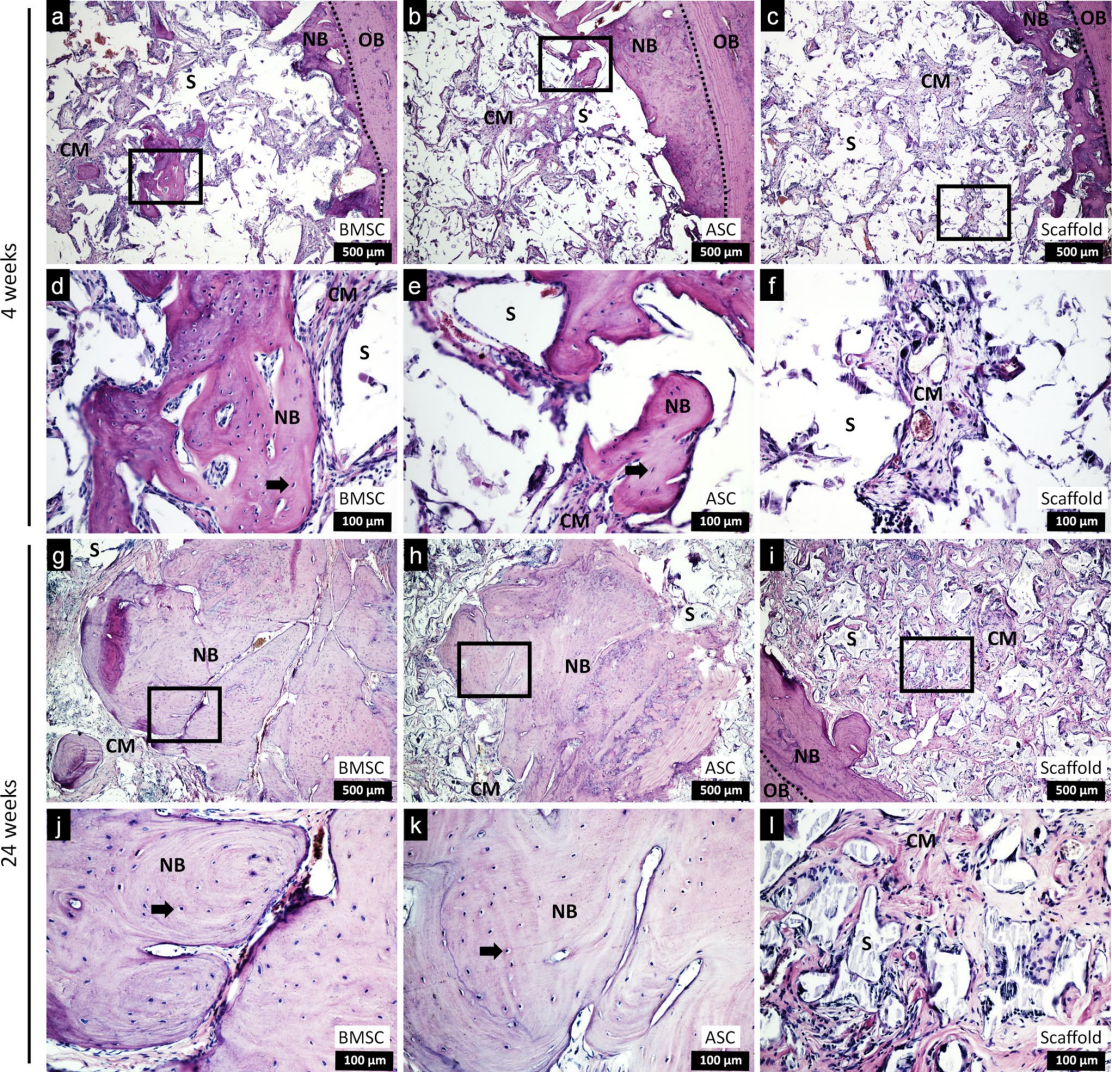
H and E staining of coronal sections of defects with scaffold/BMSC, scaffold/ASC and scaffold without cells after in vivo implantation for 4 and 24 weeks (**a**–**l**); scale bars 500 µm and 100 µm. BMSC, bone marrow mesenchymal stem cells; ASC, adipose-derived stem cells; OB, old bone; NB, new bone; CM, collagen matrix; S, scaffold; and black arrows, osteocytes

Histological evaluation by H and E staining at week 4 revealed collagen matrix formation with new bone formation, which is mainly located at the edges of the defects in the three groups. However, islands of new bone could be seen in the defects treated with scaffold/cells (Fig. 6). At week 24, considerable degradation of the scaffold was detected. Defects treated with scaffold/BMSC or scaffold/ASC showed abundant areas of mature new bone formation with osteocytes, not only at the edges, but also in the center of the defects. More non-mineralized collagen matrix formation was observed in the control defects, with new bone formation mainly along the edges of the defect.

## Discussion

The majority of previous studies have compared the bone regenerative capacity of ASC and BMSC from different donors. In this study, we examined and compared this capacity using donor-matched human ASC and BMSC. Harvesting ASC and BMSC from the same donor results in a more robust comparison by reducing the biological inter-donor variations, resulting from comparing the two types of MSC from different donors. In addition, harvesting ASC and BMSC from a homogenous group of young donors limits the possible age-related variations in the osteogenic differentiation capacity of MSC. Although limited effects of aging on this capacity have been reported (Beane et al. 2014; Siddappa et al. 2007), some studies found that aging negatively influences the properties of MSC, including the osteogenic differentiation capacity (Choudhery et al. 2014; Stolzing et al. 2008). Also, ASC and BMSC were obtained from both male and female donors. Previous studies have shown comparable osteogenic differentiation capacity of MSC obtained from male and female donors (Siddappa et al. 2007; Siegel et al. 2013). The harvested ASC and BMSC showed similar fibroblast-like morphology when cultured as monolayer and both demonstrated immunophenotype characteristics of MSC (Bourin et al. 2013; Dominici et al. 2006) with Stro-1 expression in both types of stem cells. A connection between the multipotency of MSC, especially for the osteogenic potential, and the expression of this marker has been proposed previously (Dennis et al. 2002; Rada et al. 2012). For example, Stro-1-positive BMSC demonstrated osteogenic potential confirmed by mineralization in vitro and formation of bone tissue in vivo (Dennis et al. 2002). Likewise, Stro-1-positive ASC showed osteogenic potential both in vitro and in vivo (Rada et al. 2012).

Whereas 2D culture conditions are not representative for the in vivo environment, 3D culture conditions, such as culture of cells in a 3D scaffold, offer better representation (Fitzgerald et al. 2015). Before the in vitro examination and the in vivo implantation, the attachment of ASC and BMSC on the poly(LLA-co-CL) scaffolds was confirmed. Cellular attachment and growth are affected by the physicochemical properties of the scaffold, particularly the porosity of the scaffold (Sobral et al. 2011). A scaffold that is suitable for bone tissue engineering must be highly porous with an interconnected pore structure. This provides a higher surface area for cell adhesion and eventually promotes tissue ingrowth. It was found previously that after 3 h of seeding, around 50% of MSC were attached on the poly(LLA-co-CL) scaffolds (Yassin et al. 2017). In our study, the amount of DNA in scaffolds seeded with ASC and BMSC at day 1 was at the same level. This indicates that similar number of ASC and BMSC attached to the scaffolds after seeding. Although similar trend of proliferation was observed, ASC showed higher proliferation than BMSC. This is in agreement with a previous study that found higher proliferation in ASC than donor-matched BMSC under 3D culture condition (Wu et al. 2015). However, in that study, this may be influenced by the reported higher attachment of ASC to the scaffold. 3D culture studies that compared ASC and BMSC from different donors have shown either higher proliferation of ASC than BMSC (Rath et al. 2016) or similar proliferation of the two types of MSC (Ardeshirylajimi et al. 2015).

Increased ALP activity is an early sign of in vitro osteogenic differentiation, and it is essential for initiation of mineralization of the ECM (Murshed et al. 2005), which is considered a late sign of osteogenic differentiation. Alizarin red S staining revealed mineralization and confirmed the in vitro osteogenic differentiation of 3D cultured ASC and BMSC. However, higher ALP activity was detected in BMSC. After 3 days in osteogenic medium, total ALP activity in ASC was slightly higher than in BMSC. This is most likely due to the higher number of ASC than BMSC. Although a higher number of ASC were still present after day 7, the ALP activity in BMSC was higher than in ASC, indicating that the ALP activity from each single cell of BMSC was much higher than for ASC. ALP activity has been proposed as an indicator for the in vivo bone forming capacity of the cells, since bone formation in vivo correlated with in vitro ALP activity, but not in vitro mineralization (Janicki et al. 2011). A previous study found that BMSC had greater in vitro osteogenic capacity, in terms of ALP activity and mineralization, than donor-matched ASC under both static and dynamic 3D culture conditions (Wu et al. 2015). Results from 3D cultured ASC and BMSC, obtained from different donors, have shown conflicting results, with both a higher in vitro osteogenic potential of BMSC compared with ASC (Ardeshirylajimi et al. 2015) and a lower in vitro osteogenic differentiation capacity of BMSC compared with ASC (Rath et al. 2016). Thus, harvesting MSC from different individuals may have a direct effect on the results (Mohamed-Ahmed et al. 2018).

The osteogenic capacity of ASC and BMSC was studied here both in vitro and in vivo, as the in vitro osteogenic capacity of MSC might not correlate with the capacity to form bone in vivo (Mendes et al. 2004). The calvarial defect model was selected in the current study because the structure of the calvarial bone allows the creation of standardized and reproducible defects, with adequate support for the implanted material from the underlying dura and the overlying periosteum and skin (Gomes and Fernandes 2011). It is assumed that in vivo osteogenic capacity of human MSC is enhanced when pre-differentiated in osteogenic medium prior to in vivo implantation (Ma et al. 2014). It has also been reported that the bone regenerative capacity of MSC can be promoted by chondrogenic differentiation rather than osteogenic differentiation of MSC before in vivo implantation, which results in bone formation through endochondral ossification (Brocher et al. 2013; Thompson et al. 2016). However, undifferentiated human MSC have also shown the capacity of bone regeneration in calvarial defects in rats and mice (Carvalho et al. 2014; Zong et al. 2010). This might be explained by osteogenic signal from the orthotropic environment and the underlying dura mater that stimulates MSC to form bone through intramembranous ossification (Levi et al. 2011). In contrast to the orthotropic environment, an ectopic environment lacks these stimulating osteogenic signals. In the ectopic environment, undifferentiated BMSC, but not ASC, have shown capacity to form bone, indicating intrinsic osteogenic capacity of BMSC (Brennan et al. 2017; Brocher et al. 2013). After implantation, we investigated the expression of two osteogenesis-related genes to evaluate the early osteogenic potential in vivo. Runx2 is essential for osteoblastic differentiation and synthesis of bone matrix (Long 2011), whereas collagen type I is a major protein in bone matrix involved in the mechanical properties by providing elasticity and toughness of the bone (Viguet-Carrin et al. 2006). Our results suggest that human ASC and BMSC may have actively contributed to the new bone formation, as the expression of both rat and human osteogenesis–related genes Runx2 and collagen type I was detected in the defects with scaffold/ASC or scaffold/BMSC. Nevertheless, the new bone formation might not be only attributed to the osteogenic differentiation of implanted ASC and BMSC, as paracrine signals produced by ASC and BMSC might have stimulated bone regeneration through different mechanisms (Oryan et al. 2017). For instance, implanted MSC may recruit and stimulate endogenous MSC and bone forming cells, in addition to a stimulating effect on angiogenesis. These MSC may also modulate the immune response in the defect environment to favor healing. It should be noted that the expression of the human genes in the defects with scaffold/ASC or scaffold/BMSC indicates that these cells survived during the first weeks of in vivo implantation. The survival of the implanted human cells and their contribution in the bone formation process were confirmed by detection of human nuclei embedded in the newly formed bone in defects treated with scaffold/ASC or scaffold/BMSC. This is in agreement with previous reports showing survival of human MSC in calvarial defects in immunocompromised mice (Bothe et al. 2018; Degano et al. 2008). However, in these reports, higher number of human BMSC survived compared with ASC.

In vivo imaging techniques, such as PET/CT scanning, offer a useful longitudinal non-invasive monitoring of the bone formation process (Fragogeorgi et al. 2019). Moreover, it can result in substantial reduction of the number of animals needed for different biological studies as well as the biological variability, as the same animals are examined over time (Lauber et al. 2017). The metabolic activity of the cells in the defect was monitored by PET/CT scanning that measures the uptake of the radioactive tracer into the defect site. The tracer uptake in the defects was relatively low. This might be explained by a reduction in the delivery of the tracer to the defect sites due to low vascularization of the calvarial bone (Viateau et al. 2008). The cellular activity in defects treated with scaffold/BMSC was higher than in defects treated with scaffold/ASC. This cellular activity is related not only to the implanted cells but also to the endogenous cells, meaning that more endogenous cells were recruited to the defects treated with scaffold/BMSC than those treated with scaffold/ASC. This might be due to differences between the secretome of human BMSC and ASC, which plays an important role in recruiting and stimulating the endogenous cells (Pires et al. 2016).

The defects treated with scaffold/BMSC showed accelerated and greater bone formation than defects treated with scaffold/ASC, especially at the early time-point. After 4 weeks, defects with scaffold/BMSC had significantly greater bone formation than defect with scaffold/ASC. At the later time-points (12 and 24 weeks), although the difference between defects with scaffold/BMSC and defect with scaffold/ASC was not statistically significant, defects with scaffold/BMSC showed the greatest bone formation. This indicates that the use of BMSC for bone regenerative applications results in faster and greater bone formation and, accordingly, better healing of bone defects. The superior in vivo bone forming capacity of BMSC in comparison with ASC can be linked to the higher expression of Stro-1 and ALP activity detected in vitro.

The formation of mature new bone was seen at the edges as well as in the center of the defects with scaffold/cells, unlike the control defects with poly(LLA-co-CL) scaffold without cells. Poly(LLA-co-CL) scaffold as a carrier for MSC supported in vivo bone formation as previously shown (Xing et al. 2013; Yassin et al. 2017). This scaffold is osteoconductive, but it is not bioactive in a way that stimulates osteogenesis (Yassin et al. 2017). This ensured that the active bone regeneration process was because of the MSC and not due to the scaffold. However, complete regeneration of the defects was not seen regardless of the type of the cells during our observation period. This might be explained by the relatively slow degradation of this scaffold as reported previously (Danmark et al. 2011), since degradation of the scaffold provides a space for subsequent new bone formation. Besides that, bone healing is impaired in immunodeficient animals when compared with immunocompetent animals (Rapp et al. 2016). Using different materials as scaffold, superior bone regenerative capacity of BMSC to ASC was observed in previous studies (Bothe et al. 2018; Kargozar et al. 2018; Xu et al. 2017). One of these studies compared human ASC and BMSC from the same donors using 2% hyaluronic acid hydrogel as a carrier for the cells and found that BMSC had stronger osteogenic potential than ASC both in vitro and in vivo (Xu et al. 2017). However, in that study, ASC and BMSC were from old donors and new bone formation was examined only after 6 weeks. On the other hand, other studies reported similar bone regenerative capacity of human ASC and BMSC in rats and mice (Degano et al. 2008; Jo et al. 2013; Kim et al. 2014; Wen et al. 2013). However, unlike our study, ASC and BMSC used in these studies were obtained from different donors, which may have affected the results. Apart from MSC obtained from humans, the bone regenerative capacity of ovine ASC was found to be inferior to BMSC (Niemeyer et al. 2010), but ASC and BMSC obtained from other species showed comparable bone regenerative capacity (Freitas et al. 2019; Kang et al. 2012; Lin et al. 2009; Stockmann et al. 2012).

In summary, this study compared the bone regenerative capacity of donor-matched human ASC and BMSC. These two types of MSC showed in vitro osteogenic potential. However, when these cells were implanted in calvarial defects in nude rats, BMSC showed greater capacity of bone regeneration than ASC, especially at early time-points. These results suggest that although ASC have the potential to regenerate bone, the rate of bone regeneration with ASC may be slower than with BMSC. Accordingly, BMSC are more suitable for bone regenerative applications.
